# Lipid Remodeling and Membrane Stability Contribute to Differential Chilling Tolerance in Two Dichondra (*Dichondra repens*) Genotypes

**DOI:** 10.3390/ijms27021009

**Published:** 2026-01-20

**Authors:** Sitian Liu, Junnan Lin, Jishun Jiang, Yilin Di, Xinying Liu, Zhou Li

**Affiliations:** College of Grassland Science and Technology, Sichuan Agricultural University, Chengdu 611130, China

**Keywords:** cell membrane stability, cold stress, photochemical efficiency, lipidomics, lipid peroxidation, thermophilic crop

## Abstract

Dichondra (*Dichondra repens*) is an important thermophilic Chinese herbal medicine and a key component in traditional herbal tea and beverages. It is also commonly used as an excellent ground cover plant for landscapes and cover cropping in orchards. In temperate and transition zones, thermophilic dichondra often suffers from chilling stress resulting in growth retardation and yield loss. This study aims to compare differences in photochemical efficiency, cell membrane stability, lipid peroxidation, and global lipid remodeling between two dichondra genotypes (chilling-tolerant Dr5 and chilling-sensitive Dr17) in response to a prolonged chilling stress. The results demonstrated that chilling stress significantly accelerated membrane lipid peroxidation and chlorophyll loss, resulting in reduced cell membrane stability and photochemical efficiency in two genotypes. However, Dr5 exhibits less oxidative damage, better cell membrane stability, and higher photochemical efficiency than Dr17 under chilling stress. The analysis of lipidomics found that both Dr5 and Dr17 accumulated phospholipids (Phls), glycoglycerolipids (Glls), and sphingolipids (Spls). More importantly, Dr5 exhibited 95%, 72%, 71%, 526%, 39%, 89%, 131%, 695%, or 865% increase in phosphatidic acid (PA), ceramide (Cer), hexosyl ceramide (Hex1Cer), lyso PA (LPA), lyso phosphatidylcholine (LPC), lyso phosphatidylethanolamine (LPE), lyso phosphatidylglycerol (LPG), lyso phosphatidylinositol (LPI), or lyso phosphatidylserine (LPS) content than Dr17 on day 10 of chilling stress, respectively. Dr5 also maintained significantly higher contents of PC (52%), PE (53%), PI (24%), PS (81%), PG (30%), and digalactosyl diacylglycerol (DGDG, 53%) after 20 days of chilling stress. In addition, two genotypes could maintain a stable unsaturation level of total lipids under chilling stress. These findings indicate that lipid remodeling is attributed to genetic variation in chilling tolerance of dichondra species. The current study provides an interesting data set that could be the starting point for analyzing the underlying mechanisms of chilling tolerance in thermophilic dichondra species.

## 1. Introduction

Driven by global climate change, extreme climate events including drought, rainstorm, heat stress, and low temperature are occurring with increasing frequency and intensity. Among these diverse abiotic stresses, low temperature poses a great threat to crop growth, development, and production, especially to those crops that only adapt to warm climatic conditions [1,2]. Most thermophilic crops are susceptible to chilling stress (0–15 °C) and also exhibit minimal survival rates under freezing stress (<0 °C). Dichondra (*Dichondra repens*) is widely cultivated in tropical and subtropical regions as a Chinese herbal medicine, and its extract is used to treat jaundice, dysentery, and icterohepatitis [3,4]. In addition, dichondra is also an important ground cover plant for landscaping, ecological restoration, and weed control due to its excellent ability to form a dense and low-growing lawn [5,6,7]. As a thermophilic crop, dichondra has an optimum temperature range from 25 to 30 °C for growth and development. Thus, chilling stress limits geographic distribution, yield, and promotion of dichondra [8]. During a long-term evolutionary process, plant survival and adaptation to chilling stress involve multiple mechanisms such as the accumulation of osmolytes, the biosynthesis of cold-responsive proteins, and lipid reprogramming [9]. A number of studies have also shown that plants activate signaling transduction pathways, particular gene expression, and epigenetic modification to improve the ability of chilling tolerance [10,11,12]. However, few studies have explored the response mechanism of dichondra to abiotic stress, particularly its adaptability to low-temperature stress.

The cell membrane system is sensitive to changing environmental temperature. Lipids are not only the structural basis of cell membranes but also an energy stock for stress defense [13]. When plants are subjected to abiotic stress, non-bilayer lipid structures are formed in plasma and chloroplast membranes, resulting in damage to membrane integrity and functionality [14]. Plants adjust lipid composition and unsaturation level to adapt to changing environmental conditions [15]. Phospholipids (Phls), glycoglycerolipids (Glls), and sphingolipids (Spls) are the main membrane lipids in plants. It has been found that most Phls and Spls serve as essential structural components of cellular membranes and also function as lipid signaling molecules. In contrast, the majority of glycolipids constitute integral constituents of chloroplast membranes [16,17]. Recently, high-throughput lipid profiling has provided an important technique to elucidate the individual function of large-scale different lipids in plants under stressful conditions. For example, lipid remodeling is an important mechanism for *Dendrobium catenatum* to adapt to cold stress [18]. The overexpression of polyamine-biosynthetic key gene *TrSAMDC1* or *TrSAMS* in *Arabidopsis thaliana* significantly enhanced membrane system integrity and stability associated with the accumulation of multiple Phls and Glls in leaves under heat stress [19,20]. Exogenous application of diethyl aminoethyl hexanoate effectively enhanced membrane stability by alleviating drought-induced declines in Glls, Phls, and Spls [21]. In addition to the importance of lipid content, the change in lipid unsaturation level also affects plant adaptation to various abiotic stresses. Elevated lipid unsaturation enhances membrane fluidity, whereas reduced unsaturation level diminishes membrane fluidity and permeability [22]. Under cadmium stress, the membrane lipid unsaturation level exhibited a greater reduction in Cd-tolerant cultivar Pixie than Cd-sensitive cultivar Sulky, indicating that elevated lipid saturation could attenuate Cd^2+^ permeability by enhancing lipid packing density and reducing membrane fluidity [23]. Similarly, *TrFQR1* overexpression in white clover (*Trifolium repens*) suppressed heat-induced membrane hyperfluidization via reducing lipid unsaturation, thereby stabilizing plasma membrane, chloroplast, and mitochondrial functions [24]. However, plants are apt to increase the degree of lipid unsaturation to enhance membrane fluidity when exposed to cold stress, counteracting the membrane rigidification induced by low temperature [22]. Lipid unsaturation level significantly declined due to the mutation of lipid desaturase in *Arabidopsis thaliana* resulting in the hypersensitivity to chilling stress [25,26]. Transgenic maize (*Zea mays*) plants overexpressing an *ACP2* for lipid desaturation exhibited better chilling tolerance than the wild type [27]. However, the regulatory role of lipid reprogramming and unsaturation level in conferring chilling adaptation of dichondra species remains to be further investigated.

Our earlier research comprehensively evaluated drought tolerance in 33 dichondra genotypes through an integrated assessment of leaf morphology, genetic diversity, and physiological responses. Among all accessions, wildland germplasm Dr5 was identified as the most drought-tolerant genotype associated with stable chlorophyll metabolism and photosynthetic capacity, a strong antioxidant defense system, and good osmotic adjustment under drought stress [5,7]. Further analysis revealed that Dr5 also exhibited superior chilling tolerance, which could be attributed to nitric oxide-regulated photosynthetic stability, enhanced sugar metabolism, and improved antioxidant defense [8]. These findings indicate that Dr5 represents a promising elite genotype for breeding climate-resilient dichondra cultivars adapted to abiotic stress environments. However, the role of lipid remodeling and unsaturation in underpinning the differential chilling tolerance between cold-resistant Dr5 and cold-sensitive Dr17 remains unclear. The current experiment was further conducted to investigate the differential chilling tolerance between cold-resistant Dr5 and cold-sensitive Dr17 in relation to changes in membrane lipid peroxidation, membrane system stability, global lipid remodeling, and lipid unsaturation level. Unraveling the variation in chilling tolerance between different plant genotypes is vital for breeding next-generation cold-resilient crops and deploying region-specific adaptation strategies across diverse climatic zones.

## 2. Results

### 2.1. Chilling Stress Affected Cell Membrane Stability and Lipid Peroxidation, Chlorophyll Content, and Photochemical Efficiency

**Figure 1:**
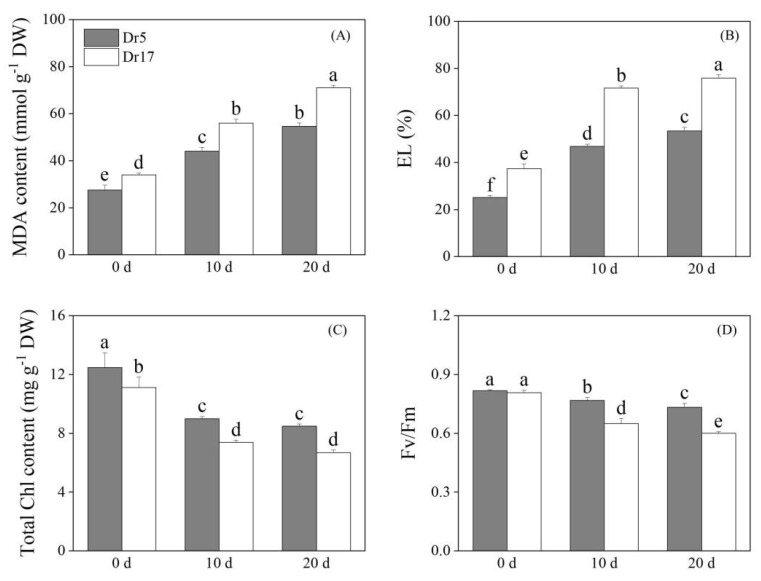
Changes in (**A**) malondialdehyde (MDA), (**B**) electrolyte leakage (EL), (**C**) total chlorophyll (Chl) content, and (**D**) the ratio of variable to maximal fluorescence (Fv/Fm) in leaves of two dichondra genotypes (Dr5 and Dr17) during chilling stress. Vertical bars represent standard errors of the mean (*n* = 4). Different small letters above columns indicate significant differences (*p* < 0.05).

Chilling stress caused a significant increase in MDA content in two genotypes (Dr5 and Dr17) (Figure 1A). However, Dr5 exhibited significantly lower MDA content than Dr17 under both normal conditions and chilling stress (Figure 1A). MDA content in leaves of Dr17 was 1.26 times higher than Dr5 after 10 days of chilling stress. Dr17 also had 1.29 times higher MDA content than Dr5 on day 20 of chilling stress (Figure 1A). Chilling stress led to a significant increase in EL for both Dr5 and Dr17, but Dr5 maintained significantly lower EL than Dr17 from 0 to 20 days of chilling stress (Figure 1B). On day10 and day 20 of chilling stress, Dr17 exhibited more than 50% higher EL than Dr5 (Figure 1B). Chilling stress induced Chl loss in leaves of two genotypes (Figure 1C). As compared to Dr17, Dr5 maintained significantly higher Chl content from 0 to 20 days of chilling stress (Figure 1C). Chl content in Dr5 was 20% greater than that in Dr17 by the 20th day of chilling stress (Figure 1C). Chilling stress also led to a gradual decrease in Fv/Fm of two genotypes, but Dr5 had an 19% and 22% higher Fv/Fm compared to Dr17 on day 10 and day 20 of chilling stress, respectively (Figure 1D).

### 2.2. Chilling Stress Affected Lipid Remodeling and Unsaturation Level in Two Dichondra Genotypes

**Figure 2:**
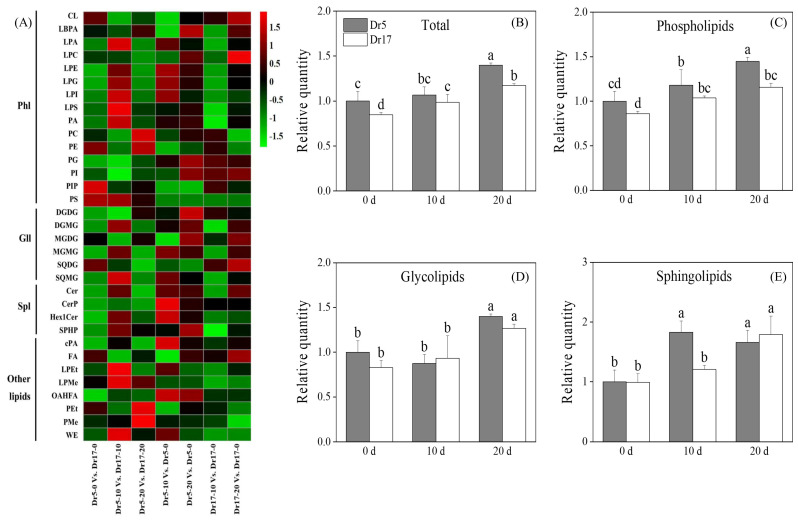
Changes in (**A**) heat map of 34 different lipid classes, (**B**) total lipid content, (**C**) phospholipid (Phl) content, (**D**) glycoglycerolipid (Gll) content, and (**E**) sphingolipid (Spl) content in leaves of two dichondra genotypes (Dr5 and Dr17) during chilling stress. Vertical bars represent standard errors of the mean (*n* = 4). Different small letters above columns indicate significant differences (*p* < 0.05).

**Figure 3:**
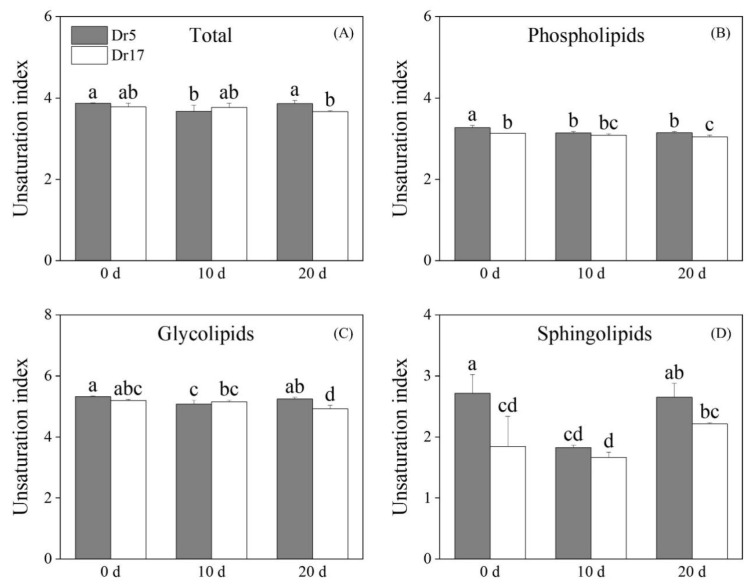
Changes in (**A**) unsaturation index of total lipid, (**B**) unsaturation index of phospholipids (Phls), (**C**) unsaturation index of glycoglycerolipids (Glls), and (**D**) unsaturation index of sphingolipids (Spls) in leaves of two dichondra genotypes (Dr5 and Dr17) during chilling stress. Vertical bars represent standard errors of the mean (*n* = 4). Different small letters above columns indicate significant differences (*p* < 0.05).

A total of 33 lipid classes were identified in leaves of two dichondra genotypes, including 15 Phls, 6 Glls, 4 Spls, and 8 other lipids (Figure 2A and Table 1). Full names and their corresponding abbreviations of 34 lipid classes are shown in Table 1. A heat map of different comparison groups including Dr5-0 vs. Dr17-0, Dr5-10 vs. Dr17-10, Dr5-20 vs. Dr17-20, Dr5-10 vs. Dr5-0, Dr5-20 vs. Dr5-0, Dr17-10 vs. Dr17-0, and Dr17-20 vs. Dr17-0 displayed that chilling stress induced lipid remodeling in two genotypes (Figure 2A). Total lipid content in leaves of two genotypes gradually increased with the prolonged chilling stress (Figure 2B). Dr5 exhibited an 18%, 8%, and 20% higher total lipid content than Dr17 on day 0, day 10, and day 20 of chilling stress, respectively (Figure 2B). Chilling stress induced a significant increase in Phl content in leaves of two genotypes, and Dr5 exhibited significantly higher Phl content than Dr17 on day 20 of chilling stress (Figure 2C). Gll content in leaves of two genotypes significantly increased after 20 days of chilling stress, but no significant difference in Gll content was detected between Dr5 and Dr17 during chilling stress (Figure 2D). Dr5 had 1.5 times higher Spl content than Dr17 on day 10 of chilling stress (Figure 2E). The total lipid unsaturation index decreased in leaves of Dr5 but did not alter in Dr17 on day 10 of chilling stress (Figure 3A). Chilling stress induced a significant decline in the Phl lipid unsaturation index of Dr5 and Dr17 (Figure 3B). The Gll unsaturation index of Dr5 decreased after 10 days of chilling stress, but the Gll unsaturation index of Dr5 decreased after 20 days of chilling stress (Figure 3C). The Spl unsaturation index of Dr5 significantly decreased on day 10 of chilling stress and recovered to a normal level on day 20 of chilling stress (Figure 3D). However, the Spl unsaturation index of Dr17 significantly increased on day 20 of chilling stress (Figure 3D).

### 2.3. Chilling Stress Affected Phospholipid Composition in Two Dichondra Genotypes

**Figure 4:**
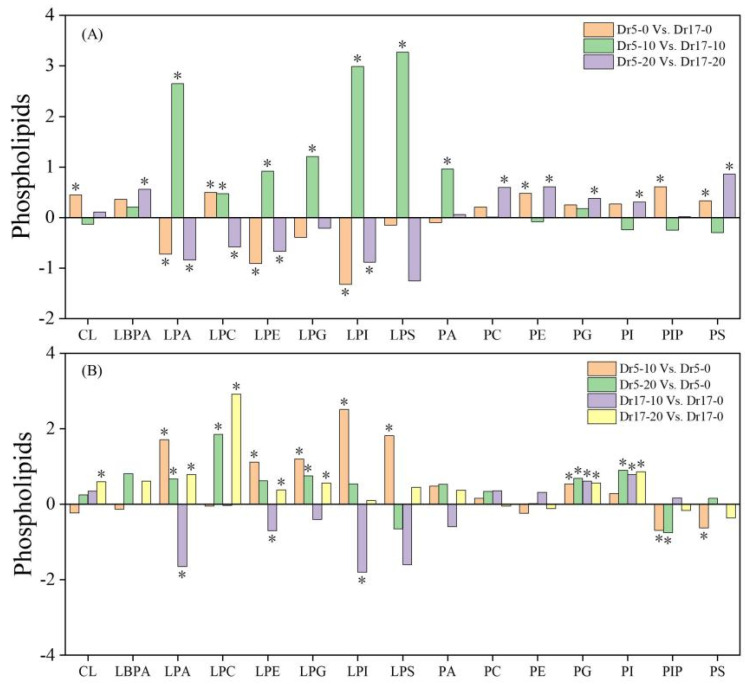
Changes in different phospholipid (Phl) classes in (**A**) Dr5-0 vs. Dr17-0, Dr5-10 vs. Dr17-10, and Dr5-20 vs. Dr17-20 and (**B**) Dr5-10 vs. Dr5-0, Dr5-20 vs. Dr5-0, Dr17-10 vs. Dr17-0, and Dr17-20 vs. Dr17-0. Asterisk (*) above each column indicates a significant difference between the two treatments (*p* < 0.05). The *X*-axis represents different Phl classes, while the *Y*-axis represents the comparison of two treatments in terms of the relative content of each Phl class.

Phl classes significantly changed in two genotypes during chilling stress (Figure 4A,B). For Dr5-0 vs. Dr17-0, Dr5 exhibited significantly higher contents of CL, LPC, PE, PIP, and PS than Dr17 on day 0 of chilling stress (Figure 4A). Dr5 had significantly higher contents of LPA, LPC, LPE, LPG, LPI, LPS, and PA than Dr17 on day 10 of chilling stress, as demonstrated by Dr5-10 vs. Dr17-10 (Figure 4A). For Dr5-20 vs. Dr17-20, significantly higher contents of LBPA, PC, PE, PG, PI, and PS, and lower contents of LPA, LPC, LPE, and LPI were detected in Dr5 compared to Dr17 on day 20 of chilling stress (Figure 4A). In response to the 10th day of chilling stress, LPA, LPE, LPG, LPI, LPS, and PG significantly increased in Dr5 (Dr5-10 vs. Dr5-0), and LPC, LPG, and PG, and PI significantly increased in Dr5 after 20 days of chilling stress (Dr5-20 vs. Dr5-0) (Figure 4B). For Dr17-10 vs. Dr17-0, most Phl classes significantly reduced or did not change in Dr17 on day 10 of chilling stress (Figure 4B). As compared to day 0, contents of CL, LPA, LPC, LPE, LPG, PG, and PI significantly increased in Dr17 on day 20 of chilling stress (Dr17-20 vs. Dr17-0) (Figure 4B).

### 2.4. Chilling Stress Affected Glycoglycerolipid Composition in Two Dichondra Genotypes

**Figure 5:**
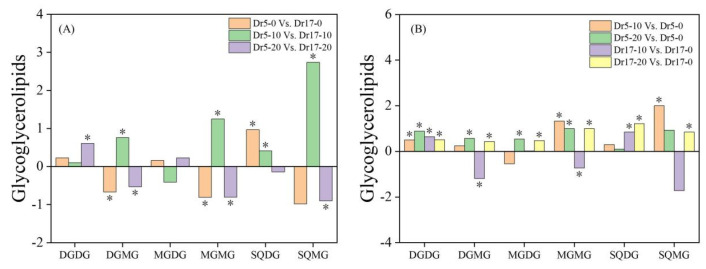
Changes in different glycoglycerolipid (Gll) classes in (**A**) Dr5-0 vs. Dr17-0, Dr5-10 vs. Dr17-10, and Dr5-20 vs. Dr17-20 and (**B**) Dr5-10 vs. Dr5-0, Dr5-20 vs. Dr5-0, Dr17-10 vs. Dr17-0, and Dr17-20 vs. Dr17-0. Asterisk (*) above each column indicates a significant difference between the two treatments (*p* < 0.05). The *X*-axis represents different Gll classes, while the *Y*-axis represents the comparison of two treatments in terms of the relative content of each Gll class.

For changes in Gll classes, Dr5 had significantly higher SODG content and lower contents of DGMG and MGMG than Dr17 on day 0 of chilling stress (Dr5-0 vs. Dr17-0) (Figure 5A). On day 10 of chilling stress, contents of DGMG, MGMG, SQDG, and SQMG in Dr5 were significantly higher than those in Dr17 (Dr5-10 vs. Dr17-10), while only the content of DGDG in Dr5 was significantly higher than that in Dr17 on day 20 of chilling stress (Dr5-20 vs. Dr17-20) (Figure 5A). On day 10 of chilling stress, contents of DGDG, MGMG, and SQMG in Dr5 significantly increased compared to day 0 (Dr5-10 vs. Dr5-0), and on day 20 of chilling stress, contents of DGDG, DGMG, MGDG, and MGMG also significantly increased in Dr5 (Dr5-20 vs. Dr5-0) (Figure 5B). For Dr17-10 vs. Dr17-0, contents of DGDG and SQDG significantly increased, whereas contents of DGMG and MGMG significantly declined (Figure 5B). Contents of DGDG, DGMG, MGDG, MGMG, SQDG, and SQMG significantly increased in Dr17 after 20 days of chilling stress (Dr17-20 vs. Dr17-0) (Figure 5B).

### 2.5. Chilling Stress Affected Sphingolipid and Other Lipid Compositions in Two Dichondra Genotypes

**Figure 6:**
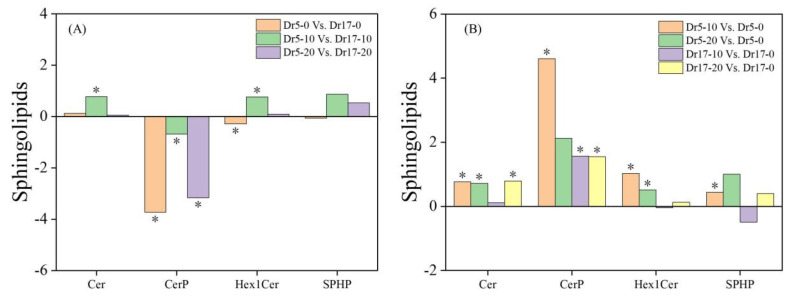
Changes in different sphingolipid (Spl) classes in (**A**) Dr5-0 vs. Dr17-0, Dr5-10 vs. Dr17-10, and Dr5-20 vs. Dr17-20 and (**B**) Dr5-10 vs. Dr5-0, Dr5-20 vs. Dr5-0, Dr17-10 vs. Dr17-0, and Dr17-20 vs. Dr17-0. Asterisk (*) above each column indicates a significant difference between the two treatments (*p* < 0.05). The *X*-axis represents different Spl classes, while the *Y*-axis represents the comparison of two treatments in terms of the relative content of each Spl class.

**Figure 7:**
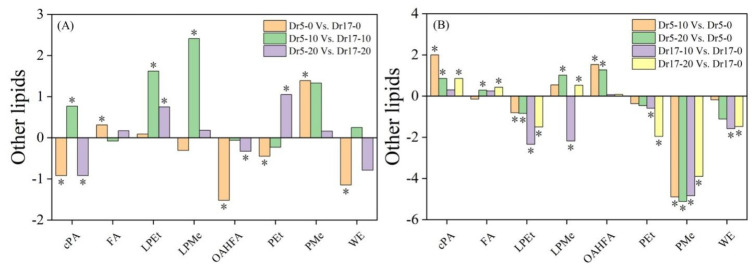
Changes in different other lipid classes in (**A**) Dr5-0 vs. Dr17-0, Dr5-10 vs. Dr17-10, and Dr5-20 vs. Dr17-20 and (**B**) Dr5-10 vs. Dr5-0, Dr5-20 vs. Dr5-0, Dr17-10 vs. Dr17-0, and Dr17-20 vs. Dr17-0. Asterisk (*) above each column indicates a significant difference between the two treatments (*p* < 0.05). The *X*-axis represents other lipid classes, while the *Y*-axis represents the comparison of two treatments in terms of the relative content of each lipid class.

**Figure 8:**
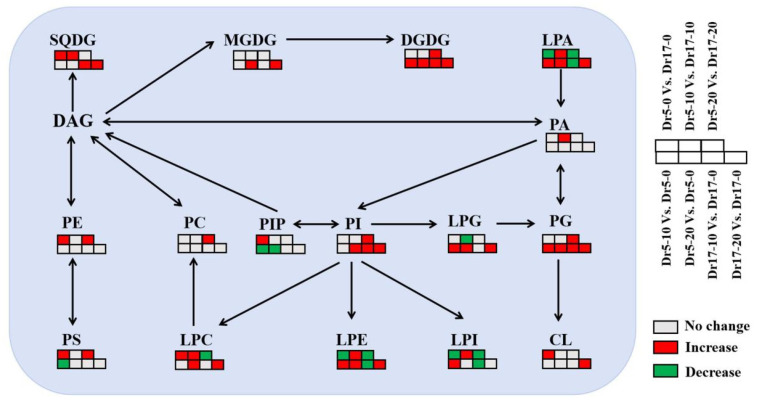
Lipid remodeling in leaves of two dichondra genotypes (Dr5 and Dr17) during chilling stress. Red, green, or gray rectangle indicates a significant increase, significant decrease, or no significant change between two treatments based on the one-way ANOVA in combination with the LSD test at the *p* < 0.05 level. Black arrows indicate potential transformations between lipids.

Dr5 exhibited significantly lower contents of CerP and Hex1Cer than Dr17 under normal conditions (Dr5-0 vs. Dr17-0), but Dr5 exhibited significantly higher contents of Cer and Hex1Cer than Dr17 on day 10 of chilling stress (Dr5-10 vs. Dr17-10) (Figure 6A). For Dr5-20 vs. Dr17-20, CerP content significantly decreased (Figure 6A). As compared to day 0, contents of Cer, CerP, Hex1Cer, and SPHP in Dr5 significantly increased on day 10 of chilling stress (Dr5-10 vs. Dr5-0), and contents of Cer and Hex1Cer also significantly improved in Dr5 on day 20 of chilling stress (Dr5-20 vs. Dr5-0) (Figure 6B). For Dr17-10 vs. Dr17-0, only CerP significantly increased (Figure 6B). For Dr17-20 vs. Dr17-0, Cer and CerP significantly increased (Figure 6B). For changes in contents of other lipids, contents of cPA, OAHFA, PEt, and WE significantly decreased, and the contents of FA and PMe significantly increased in Dr5-0 vs. Dr17-0 (Figure 7A). Contents of cPA, LPEt and LPMe significantly increased in Dr5-10 vs. Dr17-10 (Figure 7A). For Dr5-20 vs. Dr17-20, contents of LPEt and PEt significantly increased, but contents of cPA and OAHFA significantly reduced (Figure 7A). Contents of both cPA and OAHFA significantly increased in Dr5-10 vs. Dr5-0 and Dr5-20 vs. Dr5-0, and contents of other lipids significantly declined or did not significantly change in Dr17-10 vs. Dr17-0 (Figure 7B). Contents of cPA, FA and LPMe significantly increased, and contents of LPEt, PEt, PMe, and WE significantly declined in Dr17-20 vs. Dr17-0 (Figure 7B). Figure 8 shows the conversion of different lipid classes in leaves of two dichondra genotypes during chilling stress.

## 3. Discussion

Chilling stress accelerates membrane lipid peroxidation, which in turn increases membrane permeability leading to elevated EL. Therefore, a higher EL level indicates compromised membrane integrity and stability [28]. As an early-stage product of lipid peroxidation, diene conjugates (DC) serve as an important indicator for assessing early lipid peroxidation [29]. MDA is one of the main late-stage products of lipid peroxidation in plant cell membranes, and its accumulation has been regarded as the most important indicator of membrane lipid peroxidation when plants are exposed to various abiotic stresses [30,31]. Previous studies have shown that chilling stress induced a significant increase in EL and the overaccumulation of MDA, which had a negative impact on plant growth and development [32,33,34]. These findings were consistent with our current results. However, Dr5 exhibited significantly lower MDA content and EL than Dr17 during chilling stress, indicating better membrane stability of Dr5. Chilling stress not only destroys the plasma membrane but also causes serious damage to the chloroplast membrane structure, leading to impaired photosynthetic function [35,36]. Although Chl content and Fv/Fm of both dichondra genotypes showed a decreasing trend under prolonged chilling stress, Dr5 could maintain higher Chl content and Fv/Fm. These results demonstrated that both dichondra genotypes suffered from severe chilling injury, but Dr5 showed better chilling tolerance compared to Dr17.

To resist chilling injury, plants have evolved a complex mechanism to adapt to decreasing temperature primarily through membrane lipid remodeling and modulation of lipid unsaturation level [37,38]. For example, the maintenance of membrane lipid homeostasis contributed to better chilling tolerance of green bell pepper (*Capsicum annuum*) [39]. Chilling-tolerant peanut (*Arachis hypogaea*) genotype NH5 promoted total lipid content, whereas chilling-sensitive genotype FH18 reduced total lipid content in response to chilling stress [40]. Chilling stress-induced accumulation of total lipids, Phls, Glls, and Spls was observed in both dichondra genotypes, indicating that both Dr5 and Dr17 maintained lipid content to alleviate structural disorganization of cell membrane systems. Dr5 could accumulate more Phls and Spls contributing to better membrane stability than Dr17 during the same duration of chilling stress. In addition, plants elevate lipid unsaturation level to maintain membrane fluidity compromised by chilling stress, because an increased ratio of unsaturated to saturated lipids lowers the phase transition temperature of membrane lipids from liquid-crystalline to gel phase, preventing membrane rigidification under prolonged chilling conditions [13,41]. Peanut genotype NH5 with stronger chilling tolerance improved the ratio of unsaturated lipids to saturated lipids, whereas chilling-sensitive genotype FH18 had no significant change in lipid unsaturation level under chilling stress [40]. However, unsaturated lipids exhibit heightened susceptibility to ROS-induced oxidation due to allylic hydrogen abstraction [42]. The study conducted by Liu et al. found that banana (*Musa acuminata*) peel could prioritize alleviating chilling stress-induced oxidative damage by accumulating more saturated lipids and reducing the content of unsaturated lipids, because unsaturated lipids are more prone to oxidation. However, the potential impact of this lipid compositional change on membrane fluidity remains to be further elucidated [43]. In the current study, both Dr5 and Dr17 maintained stable unsaturation levels of total lipids, Phls, and Glls during chilling stress but significantly reduced the unsaturation index of Spls after 10 days of chilling stress. These findings indicated that different dichondra genotypes could maintain a stable unsaturation level to preserve the normal phase transition temperature and also reduce unsaturation levels of specific lipid classes to avoid ROS-induced oxidative damage to unsaturated lipids under chilling stress.

Remodeling membrane lipid composition represents a core adaptive strategy for plants to mitigate extreme temperature stress [44]. Spls, which are enriched in the outer layer, comprise 40% of plasma membrane lipids, regulating membrane integrity and ion permeability. Many studies have demonstrated positive roles of Phls such as PA, PC, PE, PS, PG, and PI in chilling tolerance [45]. Untargeted lipidomics revealed that methyl jasmonate alleviates chilling injury to peach fruit by promoting Phl remodeling [46]. PA acting as an important lipid second messenger regulates plant adaptation to low-temperature stress [47]. PI functions as a signaling precursor whose phosphorylation derivatives and hydrolysis products constitute the core signaling network in plant signal transduction [48]. PC and PE are the predominant structural lipids in the plasma membrane, forming its basic framework, much like the building blocks of a house. Their abundance and molar ratios modulate the formation of the Phl bilayer, thereby determining the structural integrity of the plasma membrane [49]. PG mainly accumulates in thylakoid membranes and is essential for photosystem II function due to its unique 16:1^Δ3trans^ fatty acid moiety [26]. It has been found that chilling stress reduced contents of PC and PE in banana peel, resulting in damaged plasma membranes [43]. The analysis of lipidomics confirmed the importance of PC, PE, and PG for the chilling tolerance of rice (*Oryza sativa*) plants [50]. In response to chilling stress, PA only accumulated in chilling-tolerant rice variety LJ31 but reduced in chilling-sensitive variety LD3 [51]. Japonica rice with better thylakoid membrane stability exhibited significantly higher PG than Indica rice under chilling stress [52]. In the current study, Dr5 exhibited significantly higher PA content on day 10 of chilling stress and contents of PC, PE, PI, PS, and PG after 20 days of chilling stress than Dr17. This could be one of the most pivotal reasons why Dr5 had better chilling tolerance than Dr17 associated with preferable cell membrane stability and photochemical efficiency. It has also been reported that chilling-tolerant rice variety KY131 accumulated PC, PE, and PG, which preserved plasma membrane integrity and thylakoid function. In contrast, sensitive variety DN422 reduced Phls synthesis under chilling stress [50].

In addition, Dr5 also accumulated more lysophospholipids (LPLs) including LPA, LPC, LPE, LPG, LPI, and LPS compared to Dr17 on day 10 of chilling stress. LPLs, a class of single-chain phospholipids, function as multifunctional signaling molecules in plants, participating in stress signal transduction, maintenance of membrane homeostasis, and immune regulation [53]. The accumulation of specific LPLs such as LPC, LPE, LPG, LPI, and LPS underlies the superior chilling/freezing tolerance observed in cold-adapted wheat variety compared to the sensitive one [54]. The overexpression of *GPAT* for LPA biosynthesis enhanced chilling tolerance of maize associated with better cell membrane stability [27]. Although total lipid content significantly declined in response to chilling stress, rice plants up-regulated the accumulation of PC, PE, LPC, and LPE contributing to maintenance of membrane homeostasis [51]. These findings indicate that better chilling tolerance of Dr5 could be related to LPLs remodeling.

Most Glls localize to chloroplast membrane systems including outer, inner, and thylakoid membranes, which are primarily composed of MGDG (major component), DGDG (secondary component), SQDG, and other Glls (DGMG, MGMG, and SQMG) [55,56]. DGDG, MGDG, and SQDG are the major structural lipids of the thylakoid membrane. The relative ratio of MGDG to DGDG dynamically regulates the balance between the non-bilayer and bilayer of the thylakoid membrane. This is crucial for the stability of the chloroplast membrane system under stress conditions. Meanwhile, SQDG plays a specific role in stabilizing photosynthetic protein complexes through its negative charge [57]. A recent study of Gao et al. found that Gll biosynthesis was a positive contributor to chilling tolerance of maize [27]. Chilling-tolerant rice variety KY131 improved the accumulation of MGDG under chilling stress, whereas contrary findings were found in the chilling-sensitive variety DN422 [50]. Chilling-tolerant peanut genotype NH5 exhibited higher DGDG, MGDG, and SQDG contents than chilling-sensitive FH18 [40]. In response to chilling stress, maize plant enhanced the accumulation of DGDG but reduced the accumulation of MGDG [58]. Our current findings showed that Dr5 maintained significantly higher contents of DGMG, MGMG, SQDG, and SQMG on day 10 and the content of DGDG on day 20 of chilling stress than Dr17, helping to maintain the integrity of the thylakoid membrane. Similarly, increased contents of DGDG and SQDG were conducive to the mitigation of photosynthetic damage through enhancing the integrity of the thylakoid membrane when peanut seedlings were subjected to chilling stress [40]. The accumulation of DGDG and SQDG contributed to the stability of the chloroplast membrane when rice plants suffered from chilling stress [51]. Gll remodeling could protect chloroplast membrane systems from chilling injury in dichondra and other plant species.

Although Phls and Glls have well-established roles in abiotic stress adaptation, the function of Spls in mediating stress tolerance remains understudied in plants. Notably, Cer serves as a conserved lipid signal that regulates cellular metabolism and senescence [59]. Cer, CerP, and Hex1Cer are also structural lipids in membrane systems. Cer enriches membrane fluidity; CerP electrostatically stabilizes planar regions; Hex1Cer generates curvature for stress-responsive membrane remodeling [60]. In response to chilling stress, both Dr5 and Dr17 increased the accumulation of Cer, Cerp, and Hex1Cer. Moreover, Dr5 also accumulated more Cer and Hex1Cer than Dr17 on day 10 of chilling stress. These results demonstrate that enhanced accumulation of Cer, Cerp, and Hex1Cer could improve chilling tolerance of dichondra plants. A recent study found that Cd-tolerant white clover cultivar Pixie maintained significantly higher Cer, Cerp, and Her1Cer than Cd-sensitive cultivar Sulky under Cd stress [23]. Diethyl aminoethyl hexanoate enhanced drought tolerance of white clover by upregulating accumulation of total Spls, Cer, and Hex1Cer in leaves [21]. Dr5 also exhibited significantly higher contents of other lipids such as cPA, LPEt, and Pet on day 10 or 20 of chilling stress. cPA is a cyclized derivative of PA functioning as a lipid second messenger in plants that participates in stress responses and developmental regulation [61]. However, the mechanistic basis by which cPA, LPEt and Pet confer chilling tolerance remains poorly characterized in plants. It still needs to be further investigated in the future.

## 4. Materials and Methods

### 4.1. Plant Materials and Treatments

From 21 May 2023 to 21 July 2023, two dichondra genotypes (chilling-tolerant Dr5 and chilling-sensitive Dr17) were planted in white cylindrical container (20 cm in height and 11 cm in diameter) containing sand and soil (1:1, *v*/*v*) [8]. Each genotype included 10 repetitions (10 containers), and all containers were randomly placed in the greenhouse with average temperature of 28/22 °C (day/night) and average light intensity of 700 μmol·m^−2^·s^−1^ PAR. All plants were irrigated with 1/2 Hoagland’s nutrient solution once a week [62]. After two months of establishment in greenhouse, those uniformly grown plants were placed in growth chambers for 7 days of acclimation to controlled conditions (30 °C and 700 μmol·m^−2^·s^−1^ PAR in the daytime for 14 h and 25 °C at night for 10 h). Plants were then exposed to chilling stress at 15 °C/10 °C (day/night) for 20 days. Leaves were sampled from four independent biological replications (four containers) on day 0, 10, and 20 during chilling stress.

### 4.2. Measurement of Cell Membrane Stability and Membrane Lipid Peroxidation

For the determination of electrolyte leakage (EL), 0.1 g of fresh leaf samples was washed twice in deionized water and then soaked in 100 mL of deionized water for 20 h at 24 °C. The initial conductivity of the soaking solution was measured and recorded as the C_initia_. These leaves and soaking solution were then autoclaved at 120 °C for 15 min, and the final conductivity of soaking solution was detected and recorded as C_final_ after cooling to 24 °C. The EL was calculated as the percentage of the C_initia_ in the C_final_ [63]. To determine the malondialdehyde (MDA) content, 0.1 g of fresh leaves was ground with 1.5 mL of pre-cooled phosphate buffer (50 mM and pH 7.8). The homogenate was centrifuged at 12,000× *g* for 20 min at 4 °C. The supernatant (0.5 mL) was mixed with 1 mL of reaction mixture containing 20% trichloroacetic acid and 0.5% thiobarbituric acid. The mixture was then incubated in a boiling water bath for 15 min and cooled to room temperature. After being centrifuged at 8000× *g* for 10 min, the absorbance of supernatant was determined at 532 and 600 nm [64].

### 4.3. Measurement of Chlorophyll Content and Photochemical Efficiency

To determine the chlorophyll (Chl) content in leaves, 0.1 g of fresh samples were soaked in 5 mL of dimethyl sulfoxide and stored in the dark until all leaves were colorless. The absorbance of extraction solution was determined at 645 and 663 nm. These leaves were taken out from extraction solution and dried in an oven to obtain dry weight. Total Chl content was calculated according to Barnes’s method [65]. The determination of the ratio of variable to maximal fluorescence (Fv/Fm) was performed using a Chl fluorescence system (Hansatech, King’s Lynn, UK) [66].

### 4.4. Analysis of Lipidomics

Total lipids in leaves were extracted and separated by a high-performance liquid chromatography–mass spectrometry (UHPLC-MS) system (Vanquish UHPLC/Q Exactive Plus, Thermo Fisher, Waltham, MA, USA). Fresh leaves were freeze-dried for 3 days until a consistent weight was achieved. Lyophilized leaves were ground into a fine powder on ice. Powders (20 mg) were mixed with 300 μL chloroform and methanol (*v*:*v*, 2:1) and shaken for 30 min. The mixture was then centrifuged at 12,000× *g* for 20 min, and the supernatant was transferred to a new centrifuge tube. The isopropanol (300 μL) was added to dissolve the residue. After being shaken again for 30 min, the mixture was centrifuged at 12,000× *g* for 20 min. The supernatant was collected again. The supernatants obtained from two rounds of centrifugation were pooled for lipidomic analysis by using the Vanquish UHPLC/Q Exactive Plus. Column (ACQUITY UPLC BEH C18, 100 × 2.1 mm, 1.7 μm) temperature was maintained at 55 °C. Acetonitrile/water (60:40) with 10 mM of ammonium formate and 0.1% formic acid was used as mobile phase A. Isopropanol/acetonitrile (90:10) with 10 mM of ammonium formate and 0.1% formic acid was used as mobile phase B. The gradient or flow rate was set to 95/5~0/100 within 17 min or 0.4 mL·min^−1^, respectively. Lipid species were identified using Lipidsearch 4.2 (Thermo Fisher). First, potential lipid molecular formulas were proposed based on the mass-to-charge ratio (*m*/*z*) of precursor ion. Then, the lipid structures were verified and confirmed by matching the fragment ion data against known lipid species in the built-in database [23]. The relative quantification in non-biased lipidomics was derived from the peak area of each lipid molecular species. Unsaturation index of lipids was calculated by using the formula (*n* × mol% lipid)/100 (*n* indicates the number of double bonds in the lipid molecular species and mol% indicates the composition percentage of individual lipid molecular species) [67].

### 4.5. Statistical Analysis

Significant variances in physiological and lipidomic parameters across different sampling dates were analyzed based on two-way ANOVA and the Tukey Test (*p* < 0.05). A significant increase or decrease in lipid content between the two treatments (Dr5-0 vs. Dr17-0, Dr5-10 vs. Dr17-10, Dr5-20 vs. Dr17-20, Dr5-10 vs. Dr5-0, Dr5-20 vs. Dr5-0, Dr17-10 vs. Dr17-0, and Dr17-20 vs. Dr17-0) was estimated by one-way ANOVA in combination with the least significant difference (LSD) test at the *p* < 0.05 level. Four independent biological replications were used to analyze each parameter.

## 5. Conclusions

Chilling stress accelerated membrane lipid peroxidation and also reduced cell membrane stability and Chl content in dichondra plants of two different genotypes, resulting in depressed photochemical efficiency. In response to chilling stress, both Dr5 and Dr17 accumulated Phls, Glls, and Spls. Compared with Dr17, Dr5 exhibited higher levels of Phl, Gll, and Spl under chilling stress. In addition, two genotypes could maintain a stable unsaturation level of total lipids to preserve the normal phase transition temperature and also reduce unsaturation levels of Spls to avoid ROS-induced oxidative damage to unsaturated lipids under chilling stress. These findings indicate that lipid remodeling is attributed to genetic variation in chilling tolerance of dichondra and other plant species. The cold-tolerant genotype Dr5 can serve as crucial breeding material for developing new dichondra varieties with enhanced chilling tolerance while also providing a candidate genotype for investigating the molecular mechanisms underlying cold adaptation in this species. However, these findings are derived from a single experimental batch, and the absence of batch-to-batch replication somewhat limits the universality of the conclusions. To address this, subsequent studies could validate membrane stability and lipid remodeling under field conditions over multiple winter seasons.

## Figures and Tables

**Table 1:** Abbreviation of each lipid class and their corresponding full forms.

Class	Abbreviation	Full Name
Phospholipids	CL	Cardiolipin
	LBPA	Lyso bisphosphatidic acid
	LPA	Lyso phosphatidic acid
	LPC	Lyso phosphatidylcholine
	LPE	Lyso phosphatidylethanolamine
	LPG	Lyso phosphatidylglycerol
	LPI	Lyso phosphatidylinositol
	LPS	Lyso phosphatidylserine
	PA	Phosphatidic acid
	PC	Phosphatidylcholine
	PE	Phosphatidylethanolamine
	PG	Phosphatidylglycerol
	PI	Phosphatidylinositol
	PIP	Phosphatidylinositol phosphate
	PS	Phosphatidylserine
Glycoglycerolipids	DGDG	Digalactosyl diacylglycerol
	DGMG	Digalactosyl monoacylglycerol
	MGDG	Monogalactosyl diacylglycerol
	MGMG	Monogalactosyl monoacylglycerol
	SQDG	Sulfoquinovosyl diacylglycerol
	SQMG	Sulfoquinovosyl monoacylglycerol
Sphingolipids	Cer	Ceramide
	CerP	Ceramide phosphate
	Hex1Cer	Hexosyl ceramide
	SPHP	Sphingosine phosphate
Other lipids	cPA	Cyclic phosphatidic acid
	FA	Fatty acid
	LPEt	Lyso-phosphatidylethanol
	LPMe	Lyso phosphatidylmethanol
	OAHFA	O-acyl-(gamma hydroxy) fatty acid
	PEt	Phosphatidylethanol
	PMe	Phosphatidylmethanol
	WE	Wax monoesters

## Data Availability

The original contributions presented in this study are included in the article. Further inquiries can be directed to the corresponding author.
